# Less Can Be Enough: Sustained Remission of Pediatric Eosinophilic Esophagitis With Low‐Frequency Dupilumab

**DOI:** 10.1002/ccr3.72457

**Published:** 2026-05-06

**Authors:** Giovanni Scatigna, Annarita Iadecola, Martina Piersanti, Giovanni Di Nardo, Maurizio Mennini

**Affiliations:** ^1^ NESMOS Department, Faculty of Medicine and Psychology Sapienza University of Rome, Pediatric Unit, Sant'Andrea University Hospital Rome Italy

**Keywords:** dupilumab, eosinophilic esophagitis, low‐frequency dosing, pediatric, precision medicine

## Abstract

Eosinophilic esophagitis (EoE) is a chronic, immune‐mediated disease requiring long‐term therapy. Dupilumab, an interleukin‐4 and interleukin‐13 receptor antagonist, is approved for EoE at a weekly 300 mg dosing regimen. However, data on reduced‐frequency dosing, especially in pediatric patients, remain limited. This is the case of a 12‐year‐old girl presented with vomiting, food impaction, and dysphagia. Endoscopy revealed longitudinal furrows and whitish exudates (EREFS 4), and histology confirmed EoE with 75 eosinophils/high‐power field (HPF) and high EoEHSS grade and stage scores. Conventional therapies with proton pump inhibitors, topical fluticasone, and an elimination diet achieved only partial improvement. Dupilumab (300 mg every 2 weeks) induced complete clinical, endoscopic (EREFS 0), and histologic remission (EoEHSS grade/stage 0–1, peak < 6 eos/HPF) after 6 months. Given the remission and parental concern about prolonged therapy, the dosing interval was cautiously extended to every 3 weeks as a monitored off‐label trial. Topical steroids and dietary restrictions were discontinued. The patient remained asymptomatic and in complete histologic remission for more than 2 years (as of August 2025: EREFS 0, EoEHSS 0, with a peak eosinophil count of ≤ 6 eos/HPF) without any adverse events. This case illustrates that sustained remission of pediatric EoE may be achievable with individualized, response‐based dupilumab de‐escalation. While promising, this off‐label approach requires confirmation in prospective studies before routine implementation.

## Key Clinical Message

Individualized dupilumab de‐escalation may sustain long‐term remission in pediatric eosinophilic esophagitis. This case suggests that extended dosing intervals can maintain clinical, endoscopic, and histologic control in selected patients, supporting a personalized approach to therapy where reduced treatment intensity may remain effective under careful monitoring.

## 1 Introduction

Eosinophilic esophagitis (EoE) is a chronic inflammatory disorder of the esophagus, characterized by an accumulation of eosinophils within the esophageal epithelium. It is a progressive disease that can lead to esophageal dysfunction, significantly impacting patients' quality of life and imposing a substantial burden on healthcare systems [1]. The global prevalence of EoE is estimated to be 0.5–1 case per 1000 individuals. In children, the pooled annual incidence of EoE is 6.6 cases/100,000 persons, while the overall prevalence reaches about 34 cases/100,000 children [2]. EoE is a multifactorial disease wherein the interaction of genes and environment alters the esophageal epithelial barrier, allowing for abnormal exposure to allergens (primarily foods) and other luminal components [3, 4]. The impaired barrier leads to the local release of alarmins, including the thymic stromal lymphopoietin (TSLP) and interleukin (IL)‐33, which drive the differentiation of T helper 2 (Th2) effector cells and the consequent production of Th2 cytokines (IL‐4, IL‐5, IL‐9, and IL‐13) and eosinophil recruitment. IgEs, which play a crucial role in several atopic diseases, do not have a primary role in EoE pathogenesis [5]. In the pediatric population, the clinical manifestation of EoE is heterogeneous, often characterized by nonspecific symptoms and less prominent dysphagia or esophageal remodeling compared with adults. In infants and toddlers, common manifestations include feeding difficulties, food refusal, and failure to thrive, whereas school‐aged children frequently present with abdominal pain, vomiting, and gastroesophageal reflux [6]. The diagnosis of EoE is established based on histopathological findings, defined as ≥ 15 tissue eosinophils per high‐power field (HPF) in esophageal mucosal biopsies in the absence of alternative causes, in combination with compatible clinical symptoms [7]. Current first‐line therapies for the treatment of EoE include proton‐pump inhibitors (PPIs), swallowed topical glucocorticoids, and dietary options (elemental diet, empirical elimination diet, allergy test‐based diet). PPIs used at high doses (1–2 mg/kg in pediatrics or 40 mg twice daily in adults) are the most commonly prescribed first‐line medication for EoE. They work not only by blocking acidity but also by modulating eosinophilic inflammation [8]. Clinical trials have shown a response to PPI in approximately 30%–50% of individuals with EoE [9]. PPI responders were more likely to be older, male, and have mild endoscopic findings [10]. Swallowed topical steroids, such as viscous budesonide and swallowed fluticasone, have been shown to induce and maintain remission of EoE, with response rates ranging from 65% to 90% [11]. The European Society for Pediatric Gastroenterology, Hepatology and Nutrition (ESPGHAN) recommends the use of both PPIs and topical steroids as one of the first‐line treatment options for inducing remission of EoE in children [12]. The dietary options include: the elemental diet, which consists of using an amino acid‐based formula; the targeted elimination diet, guided by standard allergy testing (including specific IgE and/or skin prick testing); and the empiric elimination diet, which involves the removal of common food antigens to induce remission of EoE, followed by stepwise reintroduction of foods with serial endoscopies to identify specific triggers. Since 30%–40% of patients fail to respond to first‐line therapy, and these therapies are associated with side effects and negative impacts on quality of life, there was a need to identify alternative therapeutic options.

In May 2022, the US Food and Drug Administration (FDA) approved Dupilumab, a fully human monoclonal antibody, for the treatment of EoE in patients 12 years and older, making it the first FDA‐approved drug for this indication. Dupilumab inhibits signaling of IL‐4 and IL‐13, cytokines known to play key roles in EoE. It is now used as a step‐up therapy in patients who are refractory to current treatment, require continuous therapeutic cycles, or experience adverse effects from first‐line therapy. Dupilumab could be considered as a first‐line treatment in patients with multiple comorbid atopic conditions, including asthma, atopic dermatitis, and nasal polyps [13]. Randomized controlled trials of dupilumab in EoE demonstrate high efficacy in inducing histologic remission and improving symptoms and endoscopic findings among adults and children [6, 7, 8, 9, 10, 11, 12, 13, 14]. The currently approved and recommended dose of Dupilumab for the treatment of EoE is 300 mg administered weekly. We report the case of a 12‐year‐old girl with EoE, who demonstrated an excellent clinical, endoscopic and histologic response to a lower dosing regimen of Dupilumab (300 mg every 3 weeks) compared with the standard weekly schedule.

## 2 Case History

The patient, a 12‐year‐old girl at symptom onset, was first referred to our gastroenterology outpatient clinic in March 2022 with vomiting, food impaction, and typical dysphagia.

The patient has a grass pollen allergy causing seasonal rhinoconjunctivitis, treated with antihistamines as needed, and a history of atopic dermatitis since childhood, unresponsive to traditional topical therapies.

Endoscopic evaluation in April 2022 revealed longitudinal furrows and whitish exudates involving the middle and distal esophagus, corresponding to an endoscopy revealed longitudinal furrows and whitish exudates (EREFS) score of 4 (edema 1, rings 0, exudates 1, furrows 2, strictures 0). Histological examination revealed normal duodenal mucosa, mild chronic nonatrophic gastritis ( *Helicobacter pylori* negative), and esophageal biopsies with dense eosinophilic infiltration (75 eosinophils per HPF), eosinophilic microabscesses, and basal zone hyperplasia. According to the EoEHSS, both grade and stage scores were high, consistent with active EoE.

## 3 Differential Diagnosis, Investigations and Treatment

Initial treatment with lansoprazole (March–April 2022) resulted in poor symptom control. Repeat endoscopy in April 2022 again revealed longitudinal furrows and whitish spots in the distal esophagus, and histology confirmed EoE (> 15 eosinophils/HPF). Therapy was switched to topical fluticasone (125 μg, two puffs twice daily, April–July 2022), but only partial clinical improvement was achieved, with persistent vomiting. A nutritional approach with a cow's milk protein elimination diet was also attempted, without clinical benefit. A third endoscopy in July 2022 revealed ongoing basal layer cell hyperplasia and dense eosinophilic infiltration (40 eosinophils/HPF) in the distal esophagus, while the proximal esophagus remained histologically unremarkable. Symptoms improved but did not fully resolve.

In September 2022, due to persistent vomiting, the patient was hospitalized for further investigations. The work‐up included an ophthalmological evaluation with funduscopy, an intestinal transit X‐ray, an EEG during wakefulness, a contrast‐enhanced brain MRI, and an abdominal/pelvic MRI, all of which were unremarkable. Allergen testing confirmed grass pollen sensitization (specific IgE 25 kUA/L). In October 2022, treatment with oral viscous budesonide (2 mL vial, 0.25 mg/mL) was initiated, administered twice daily, resulting in significant improvement and allowing tapering to one vial twice daily in December 2022. Despite this, by February 2023, the patient continued to experience about one vomiting episode per month, along with persistent moderate–severe atopic dermatitis. Dupilumab therapy (300 mg every 2 weeks) was then started, in line with current recommendations for atopic dermatitis and continued until August 2023.

## 4 Conclusion and Results

**FIGURE 1:**
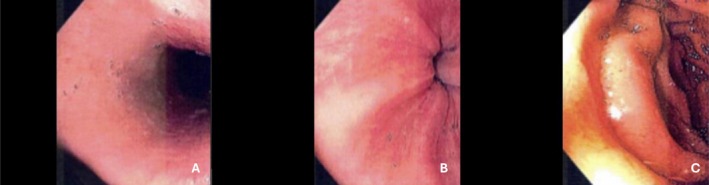
Endoscopic appearance of the upper gastrointestinal tract after low‐dose dupilumab therapy, showing normalization of mucosal findings in the proximal esophagus (A), distal esophagus (B), and second portion of the duodenum (C).

After 6 months of dupilumab, follow‐up endoscopy in September 2023 showed a completely normal esophageal mucosa (EREFS score = 0) (Figure 1), confirming full endoscopic remission. Histological reassessment demonstrated a marked improvement in both grade and stage EoEHSS scores, with a peak eosinophil count of less than six eosinophils per HPF and only minimal residual epithelial changes, consistent with near‐complete histological remission. The patient was asymptomatic. Given the remission and parental concern about the prolonged duration of therapy, the dupilumab dosing interval was cautiously extended to 300 mg every 3 weeks as a trial, resulting in sustained clinical and histological remission. This interval extension represented an off‐label use, which was thoroughly discussed with the patient's parents and undertaken with informed consent.

At her most recent follow‐up in August 2025, the patient remained in stable remission, with normal bowel and urinary function, no diarrhea, and tolerance of a free diet. She continued on dupilumab every 3 weeks. Endoscopy performed in August 2025 confirmed a macroscopically normal esophageal mucosa and complete histologic remission. The patient was regularly monitored for known dupilumab‐related adverse events, including conjunctivitis, injection‐site reactions, eosinophilia, and infections; none were observed throughout the follow‐up period.

## 5 Discussion

All current approvals (FDA, EMA) support weekly dosing in EoE; evidence for less frequent schedules is currently lacking, particularly in pediatric patients. In recent years, Dupilumab has emerged as one of the most effective therapies for EoE. Randomized clinical trials in both adolescents and adults have demonstrated significant histologic, endoscopic, and symptomatic improvements with a standard regimen of 300 mg weekly [7]. Current guidelines recommend weekly administration as the preferred option; however, in clinical practice, some patients receive biweekly dosing, particularly when treated concurrently for asthma or atopic dermatitis [13]. Evidence supporting less frequent regimens remains scarce, especially in pediatric patients. In our case, the patient achieved complete remission with every 2‐week administration and then maintained complete remission with biweekly administration, subsequently maintaining remission with dosing every 3 weeks—a schedule that differs from current protocols.

Extending the interval between dupilumab doses may offer several advantages. From a practical perspective, fewer injections can improve adherence, particularly during adolescence, when the burden of frequent medication can be a significant barrier [4]. Moreover, reduced cumulative drug exposure may lower the risk of adverse events, although dupilumab has shown a favorable safety profile in both adults and children [6]. Economic considerations are also relevant: less frequent dosing translates into substantial cost savings for families and healthcare systems, a crucial factor in managing a chronic condition that requires long‐term therapy. Finally, our case suggests that once clinical and histological remission is achieved, some patients may not require maximum treatment intensity to maintain disease control. This aligns with the principles of precision medicine, which aim to tailor therapy to individual responses rather than applying a uniform approach [9].

It is essential to acknowledge the limitations inherent in a single‐patient report. The natural history of EoE is heterogeneous, and treatment responses can vary significantly among individuals [2]. We cannot exclude the possibility that spontaneous fluctuations in disease activity contributed to the favorable outcome observed. Moreover, no validated biomarkers are currently available to guide dupilumab de‐escalation, making it challenging to identify patients who may benefit from extended dosing intervals [8]. Further studies and clinical trials are needed to systematically evaluate the efficacy, safety, and durability of lower‐frequency regimens for maintaining remission in EoE [7]. Nevertheless, this case provides preliminary evidence that sustained remission may be achievable even with reduced dosing intensity. A key strength of our report is its contribution to the scarce pediatric literature on dupilumab de‐escalation in EoE, offering a valuable perspective for the clinical management of carefully selected patients.

This case supports the feasibility of individualized, response‐based dupilumab interval extension in pediatric EoE, under strict clinical, endoscopic, and histologic monitoring. However, given the off‐label nature of this approach and the lack of controlled data, larger prospective studies are warranted before such de‐escalation can be routinely recommended.

## Author Contributions

**Giovanni Scatigna:** data curation, writing – original draft, writing – review and editing. **Annarita Iadecola:** data curation, writing – original draft, writing – review and editing. **Martina Piersanti:** data curation, writing – original draft, writing – review and editing. **Giovanni Di Nardo:** supervision, validation. **Maurizio Mennini:** conceptualization, supervision, validation.

## Funding

The authors have nothing to report.

## Ethics Statement

As a single‐case report with the patient's signed consent, no other ethics review was required.

## Consent

Written informed consent was obtained from the patient for the publication of this case report.

## Conflicts of Interest

The authors declare no conflicts of interest.

## Data Availability

The data used in this article are available upon request from the authors.
